# Evaluation of a LASSO regression approach on the unrelated samples of Genetic Analysis Workshop 17

**DOI:** 10.1186/1753-6561-5-S9-S12

**Published:** 2011-11-29

**Authors:** Wei Guo, Robert C Elston, Xiaofeng Zhu

**Affiliations:** 1 Department of Epidemiology and Biostatistics, Case Western Reserve University, 10900 Euclid Ave, Cleveland, OH 44106, USA; 2 Key Laboratory for Applied Statistics of MOE and School of Mathematics and Statistics, Northeast Normal University, Changchun 130024, China

## Abstract

The Genetic Analysis Workshop 17 data we used comprise 697 unrelated individuals genotyped at 24,487 single-nucleotide polymorphisms (SNPs) from a mini-exome scan, using real sequence data for 3,205 genes annotated by the 1000 Genomes Project and simulated phenotypes. We studied 200 sets of simulated phenotypes of trait Q2. An important feature of this data set is that most SNPs are rare, with 87% of the SNPs having a minor allele frequency less than 0.05. For rare SNP detection, in this study we performed a least absolute shrinkage and selection operator (LASSO) regression and *F* tests at the gene level and calculated the generalized degrees of freedom to avoid any selection bias. For comparison, we also carried out linear regression and the collapsing method, which sums the rare SNPs, modified for a quantitative trait and with two different allele frequency thresholds. The aim of this paper is to evaluate these four approaches in this mini-exome data and compare their performance in terms of power and false positive rates. In most situations the LASSO approach is more powerful than linear regression and collapsing methods. We also note the difficulty in determining the optimal threshold for the collapsing method and the significant role that linkage disequilibrium plays in detecting rare causal SNPs. If a rare causal SNP is in strong linkage disequilibrium with a common marker in the same gene, power will be much improved.

## Background

With the rapid development of technologies, more and more single-nucleotide polymorphisms (SNPs) have become available and, in particular, most of the rare variants can be identified using the next-generation sequencing technique. However, detecting associated rare variants that contribute to phenotypic variation is still a huge challenge. Current approaches for testing rare variants include grouping the rare variants based on a threshold of the minor allele frequency (MAF) [1], summing the rare variants weighted by the allele frequencies in control subjects [2,3], and clustering rare haplotypes using family data [4]. Another approach is to use a penalized regression, which can avoid the singular design matrix that may result from rare variants by adding a penalty, such as the least absolute shrinkage and selection operator (LASSO) and ridge penalties [5,6]. In this analysis, we evaluated the LASSO regression, linear regression and the collapsing methods by comparing their power and false positive rates. Based on the results, we recommend the LASSO approach to detect rare SNPs.

## Methods

### Data checking

In the Genetic Analysis Workshop 17 (GAW17) simulated data set, there are no missing genotype data. Among all the 24,487 SNPs, 91% have a MAF less than 0.1, 87% have a MAF less than 0.05, and 75% have a MAF less than 0.01. Moreover, 39% of the SNPs have a MAF less than 0.001, which leads to 9,433 SNPs being singletons among 697 unrelated individuals. Owing to the rareness of the variants, we do not examine Hardy-Weinberg disequilibrium as a quality control procedure in this study. Thus we include all SNPs and all individuals for the association analysis.

### LASSO regression

To deal with the singular matrix in linear regression caused by the rare variants, we adopt a statistical method that effectively shrinks the coefficients of unassociated SNPs and reduces the variance of the estimated regression coefficients. Here, we apply the LASSO penalty [7] to implement this regression analysis.

At the *i*th SNP site, we code the genotype as 0, 1, or 2 to represent 0, 1, or 2 copies of the minor allele, which is the *i*th column in the design matrix represented by *X*. For the quantitative trait *y*, the regression can be written:(1)

where *β* is the vector of regression coefficients. In a LASSO regression, the elements of *β* are the estimates that minimize the loss:(2)

where *n* is the number of individuals, *L* is the number of SNP sites, and *λ* is the tuning parameter. The LASSO regression was implemented in the R package glmnet.

### Gene-level association tests

The association is tested on the gene level. Within a gene, the dependent variable is Q2 of the GAW17 data set, and the independent variables are the genotypes of all the SNPs in the gene. We use a model, with a LASSO penalty, in which no interactions are involved. This model is indexed as M1. To test for the association between a gene and Q2, we use *F* statistics to test for the significance between models M1 and M0, where M0 is taken to be the model under the null hypothesis that *β* is a vector of zeros. Let RSS_M1_ and RSS_M0_ be the residual sums of squares of models M1 and M0, respectively. To correct for selection bias, we use the generalized degrees of freedom (GDF) [8], indicated by GDF(*M*), in the *F* tests for model M1; the GDF is larger than the number of nonzero coefficients. The *F* statistic is constructed as follows:(3)

which asymptotically follows the *F* distribution with (GDF(*M*) – 1,Â *n* – GDF(*M*)) degrees of freedom. The *P*-values for each gene are obtained from the *F* distribution given in Eq. (3).

GDF and *λ*

In classical linear models, the number of covariates is fixed; therefore the number of degrees of freedom is equal to the number of covariates. However, the situation is different in a LASSO regression: The number of nonzero coefficients can no longer accurately measure the model complexity. For a LASSO regression, which involves variable selection, the GDF was introduced [8] to correct for selection bias and to accurately measure the degrees of freedom of the obtained model. The GDF of a model is defined as the average sensitivity of the fitted values to a small change in the observed values. The parametric bootstrapping method is used to estimate the GDF [8,9].

Suppose that the observed value *y_i_*, *i* = 1, …, *n*, is modeled as *μ_i_* + *ε*, where *μ_i_* is the expectation of *y_i_* and *ε* is Gaussian white noise with variance *σ*^2^. An estimate *s*^2^ for *σ*^2^ can be obtained by an ordinary regression. Given a modeling procedure *M*: *y* → *μ*, GDF(*M*), the GDF of the modeling procedure *M*, can be estimated as follows: (1) For *t* = 1, …, *T*, where *T* = 100 here, first generate *ε_ti_* ~ normal(0, *s*^2^), *i* = 1, …, *n*. Then, evaluate  on the basis of the modeling procedure. (2) Calculate  as the regression slope from:(4)

(3) Finally, calculate:(5)

Given GDF(*M*), the extended Akaike’s A Information Criterion (AIC) is defined as:(6)

Thus the tuning parameter *λ* is selected to be the one that minimizes the extended AIC value.

### Alternative methods: *F*_linear_ and combined multivariate and the collapsing method for quantitative traits

As a comparison, we also carry out the *F* test based on general linear regression for each gene, which we call *F*_linear_. A second alternative method is the combined multivariate and collapsing (CMC) method [1], which is a unified approach that combines collapsing and multivariate tests for a binary trait. We modify the CMC method for the quantitative trait, in which markers are divided into rare and common subgroups, on the basis of a predefined allele frequency threshold (*δ*); within the rare subgroup an individual is coded 1 if a rare allele is present at any of the variant sites and 0 otherwise. After this collapsing, we calculate the *F* test to test for the association. We call this approach QCMC(*δ*) for convenience, and we consider *δ* = 0.01 and 0.05 in this paper.

## Results

We evaluated the power and false-positive rates of the *F*_LASSO_, *F*_linear_, QCMC(0.01), and QCMC(0.05) tests based on the 200 replicates of the GAW17 data set. The significance level of the tests was first set to 1.6 × 10^–5^, which is the Bonferroni-corrected significance level of 0.05 adjusted by the number of genes, that is, 0.05/3,205. However, because of the small sample sizes in the GAW17 data set, the power of the association tests was poor and could not be compared in our four tests. Therefore we also used the weak significance level of 0.01 for method comparison.

We examined the answers to the GAW17 simulation after our association analyses were completed. In the answers, Q2 is influenced by 72 SNPs in 13 genes, where the MAFs and effect sizes (*β_i_*, the elements of *β*) could be found for each causal SNP. Thus the variance contributed by each SNP to the phenotype could be calculated as  under the assumption of an additive model, where *q* is the MAF. Therefore we calculated the variance contribution for a gene using:(7)

As shown in Table 1, both genes *VNN3* and *VNN1* have a variance contribution of approximately 0.02; *SREBF1*, *BCHE*, *VLDLR*, *SIRT1*, *PDGFD*, *LPL*, and *PLAT* have variance contributions of approximately 0.01 individually; and *RARB*, *GCKR*, *VWF*, and *INSIG1* have variance contributions between 0.0002 and 0.005. The power is dependent on the variance attributed to the gene.

**Table 1:** True variance contributions of 13 causal genes given in the GAW17 answers

	*VNN3*	*VNN1*	*SREBF1*	*BCHE*	*VLDLR*	*SIRT1*	*PDGFD*	*LPL*	*PLAT*	*RARB*	*GCKR*	*VWF*	*INSIG1*
Number of SNPs	15	7	24	29	27	24	11	20	29	11	1	8	5
Number of causal SNPs	7	2	10	13	8	9	4	3	8	2	1	2	3
Average MAF of the causal SNPs	0.0206	0.0882	0.0022	0.0010	0.0013	0.0012	0.0029	0.0060	0.0021	0.0029	0.0122	0.0032	0.0007
Variance contribution	0.0239	0.0193	0.0125	0.0115	0.0111	0.0100	0.0098	0.0097	0.0090	0.0048	0.0034	0.0021	0.0002

**Figure 1:**
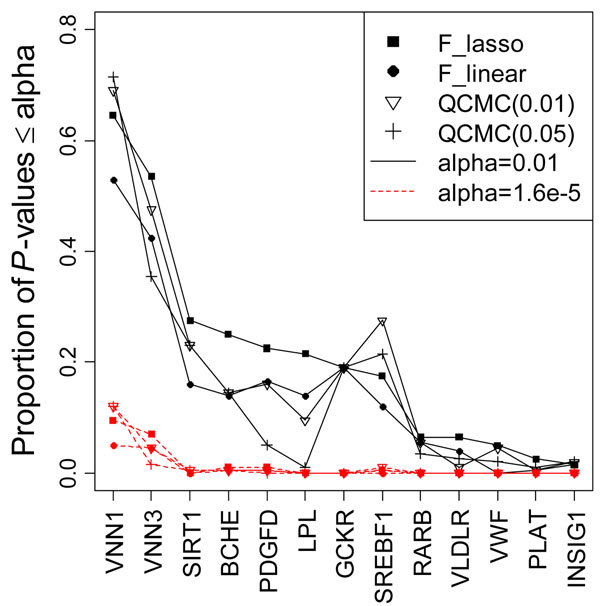
**Power to detect 13 causal genes at the significance levels of 0.01 and 1.6 × 10^–5^ in 200 replicates.** The *x*-axis indicates the 13 genes sorted in decreasing order of the power of the *F*_LASSO_ test, and the *y*-axis indicates the corresponding power. The power is shown as solid black lines for the significance level 0.01 and as red dashed lines for the significance 1.6 × 10^–5^.

We evaluated the power of the four methods based on the 13 causal genes using the 200 replicates (Figure 1). In general, the LASSO regression outperformed linear regression for all causal genes and gained more than 10% power on the first four genes, as shown in Figure 1. The QCMC(0.01) method performed better than the QCMC(0.05) method because 91.7% of the MAFs of causal SNPs were less frequent than 0.01. Except for the *VNN1* and *SREBF1* genes, the LASSO method was more powerful than the two QCMC methods. This is quite easy to understand. The *VNN1* gene has two causal SNPs, which have MAFs of 0.006 and 0.17, and all the causal SNP variants are less frequent than 0.005 in the *SREBF1* gene. For this reason, both the QCMC(0.01) and the QCMC(0.05) tests are able to collapse the causal SNPs perfectly and thereby lead to a higher power than the LASSO approach for these two genes.

**Figure 2:**
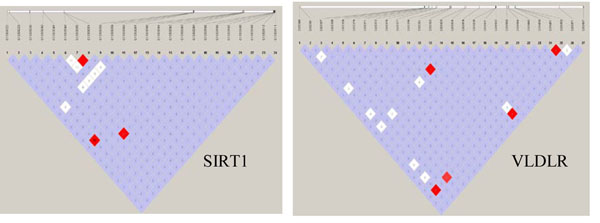
**Linkage disequilibrium plot for genes *SIRT1* and *VLDLR.*** Linkage disequilibrium plots generated from Haploview. The values of *R*^2^ are shown in each cell. The color code in the Haploview plot follows the standard color scheme for Haploview: white, |*D*′| < 1, LOD < 2; shades of pink/red, |*D*′| < 1, LOD ≥ 2; blue, | *D*′| = 1, LOD < 2; red, |*D*′| = 1, LOD ≥ 2.

In general, all the tests increased the power when a gene’s contribution to the phenotype variation increased. However, we observed some exceptions, possibly because the power depends on many other factors, such as allele frequency and linkage disequilibrium among the SNPs within a gene. First, although their contributions to the phenotype variation were similar, we had more power to detect *VNN1*, which consists of two causal SNPs, with one of them being common (MAF = 0.17), than *VNN3*, which consists of seven rare causal SNPs. Second, for the *GCKR* gene, which has only one causal SNP, we also had reasonable power, in contrast to its small contribution to the phenotype variation. The association for these two genes was concentrated in a small number of causal SNPs and hence was easier to detect. Third, the *SIRT1* and *VLDLR* genes had a similar number of SNPs, number of causal SNPs, MAFs, and variance contribution; however, *SIRT1* gained much more power than *VLDLR* did. To understand why, we examined the linkage disequilibrium among each of these genes (using Haploview, http://www.broad.mit.edu/mpg/haploview) (Figure 2). *SIRT1* includes a common SNP, C10S3059 (MAF = 0.167), that is in linkage disequilibrium with the causal rare SNP, C10S3048 (MAF = 0.002). The four gametes formed by these two SNPs are CT (83.7%), CC (16.6%), GC (0.1%), and GT (0.1%); and the *D*′ value is 0.5 (*R*^2^ = 0.003). Among the 55 significant tests of the *SIRT1* gene in 200 replicates, 81.8%, 60%, and 50.9% of the LASSO models selected SNP C10S3059, C10S3048, or both, respectively, in their M1 models. However, for *VLDLR*, although SNP C9S341 (MAF = 0.095) was also in linkage disequilibrium with the causal SNP C9S444, which has MAF = 0.001 (*D*′ = 0.384 and *R*^2^ = 0.002), it was not as common as C10S3059 and the linkage disequilibrium pattern was not the same as that for *SIRT1*.

We also investigated the false-positive rates by counting the frequency of the *P*-values that were not larger than a specific significance level for all of the 3,192 noncausal genes over the 200 replicates (Table 2). For some unknown reason, all four methods had inflated false-positive rates, and the inflation of the *F*_LASSO_ test was slightly bigger than that of the other three tests, but not significantly so.

**Table 2:** False-positive rates at the significance levels of 0.01 and 1.6 × 10^–5^ (the Bonferroni-corrected significance level of 0.05)

Significance level	*F*_LASSO_	*F*_linear_	QCMC(0.01)	QCMC(0.05)
0.01	0.02793	0.02094	0.02195	0.02233
1.60 × 10^–5^	0.00016	0.00011	0.00011	0.00013

## Discussion and conclusions

In this study, we used the LASSO regression and calculated the GDF for the *F* tests to avoid selection bias. This method requires using a parametric bootstrap to obtain the GDF; therefore it is computationally not as fast as the linear regression and collapsing methods. In general, the *F*_LASSO_ test is more powerful than the other methods.

Linear regression is the least powerful approach because of the large number of rare SNPs and because no deduction is made in the large number of degrees of freedom. The collapsing test requires specifying the predefined allele frequency threshold for grouping rare SNPs. It is difficult to determine this criterion optimally when in reality the true disease model is never known. For an extreme example, the QCMC(0.001) test was identical to the linear regression approach and the QCMC(0.1) test had no power at all in these data. Therefore, from this point of view, we recommend the LASSO approach for detecting rare SNPs.

Based on the power comparison of the *SIRT1* and *VLDLR* genes, we observed some evidence that linkage disequilibrium played a significant role in detecting rare causal SNPs. If a rare causal SNP is in strong linkage disequilibrium with a common marker in the same gene, it will perform much better in terms of power. It would be of interest to further investigate the role of linkage disequilibrium between common noncausal markers and rare causal SNPs on the power to detect rare causal SNPs and hence determine a more powerful test.

## Competing interests

The authors declare that there are no competing interests.

## Authors’ contributions

WG carried out the data analysis and drafted the manuscript. RCE and XZ participated in the design of the study and coordination and edited the manuscript. All authors read and approved the final manuscript.
